# Abnormal Emotional Processing and Emotional Experience in Patients with Peripheral Facial Nerve Paralysis: An MEG Study

**DOI:** 10.3390/brainsci10030147

**Published:** 2020-03-04

**Authors:** Mina Kheirkhah, Stefan Brodoehl, Lutz Leistritz, Theresa Götz, Philipp Baumbach, Ralph Huonker, Otto W. Witte, Gerd Fabian Volk, Orlando Guntinas-Lichius, Carsten M. Klingner

**Affiliations:** 1 Biomagnetic Center, Jena University Hospital, 07747 Jena, Germany; Mina.Kheirkhah-Rahimabadi@med.uni-jena.de (M.K.); stefan.brodoehl@med.uni-jena.de (S.B.); THERESA.GOETZ@med.uni-jena.de (T.G.); Ralph.Huonker@med.uni-jena.de (R.H.); 2 Hans Berger Department of Neurology, Jena University Hospital, 07747 Jena, Germany; Otto.Witte@med.uni-jena.de; 3 Institute of Medical Statistics, Computer and Data Sciences, Jena University Hospital, 07740 Jena, Germany; lutz.leistritz@med.uni-jena.de; 4 Department of Anesthesiology and Intensive Care Medicine, Jena University Hospital, 07747 Jena, Germany; Philipp.Baumbach@med.uni-jena.de; 5 Department of Otorhinolaryngology, Jena University Hospital, 07747 Jena, Germany; fabian.volk@med.uni-jena.de (G.F.V.); orlando.guntinas@med.uni-jena.de (O.G.-L.)

**Keywords:** classification, emotion, facial nerve paralysis, LASSO, MEG

## Abstract

Abnormal emotional reactions of the brain in patients with facial nerve paralysis have not yet been reported. This study aims to investigate this issue by applying a machine-learning algorithm that discriminates brain emotional activities that belong either to patients with facial nerve paralysis or to healthy controls. Beyond this, we assess an emotion rating task to determine whether there are differences in their experience of emotions. MEG signals of 17 healthy controls and 16 patients with facial nerve paralysis were recorded in response to picture stimuli in three different emotional categories (pleasant, unpleasant, and neutral). The selected machine learning technique in this study was the logistic regression with LASSO regularization. We demonstrated significant classification performances in all three emotional categories. The best classification performance was achieved considering features based on event-related fields in response to the pleasant category, with an accuracy of 0.79 (95% CI (0.70, 0.82)). We also found that patients with facial nerve paralysis rated pleasant stimuli significantly more positively than healthy controls. Our results indicate that the inability to express facial expressions due to peripheral motor paralysis of the face might cause abnormal brain emotional processing and experience of particular emotions.

## 1. Introduction

The human facial expressions are an essential part of communication. Eyes, mouth, and brows specific movements can show emotions that are universally understandable [1,2]. It is believed that facial expressions have a direct influence on subjective feelings, so that facial expressions strengthen our emotions while suppression of that weakens emotions [3]. This theory is called the facial feedback hypothesis (FFH), and Charles Darwin [4] was one of the first who suggested that. Several studies have supported the FFH. As reported earlier [5], receptors in the facial skin return information to the brain, and when this feedback attains consciousness, it is perceived as emotion. Izard [6,7] also argued that central neural activity in the brain stem, limbic cortex, and hypothalamus is activated by the perception of an emotional stimulus, and then a signal is sent from the hypothalamus to the facial muscles and subsequently to the brain stem, hypothalamus, limbic system, and thalamus. This concept is consistent with recent studies suggesting that deliberate imitation of facial expressions is linked to neuronal activation in limbic regions such as the amygdala [8,9,10,11], which is connected to the hypothalamus and brain stem regions [12].

As a result of the above conceptual discussion, facial expressions generate a feedback cycle to the brain, but what happens to this feedback cycle if a person cannot perform the facial expressions? The inability to perform facial expressions due to the facial nerve injury is called facial nerve paralysis [13]. The facial nerve is implicated in the control of facial asymmetries and expressions [14,15], which is mainly associated with the primary sensorimotor area [16,17]. The paralysis of the facial nerve leads to loss of facial movement feedback and breaks the integrity of the sensorimotor circuit, which results in impaired connectivity within the cortical facial motor network [18,19,20,21]. Such consequences of facial nerve paralysis raise the question of whether there are differences between the processing of emotional stimuli of healthy controls and patients with facial nerve paralysis. To answer this question, we consider one of the formulations of the FFH, the *necessity hypothesis*, which states that facial expressions are “necessary to produce emotional experience” [22]. If the “*necessity hypothesis*” is correct, a person who has total facial paralysis should not experience emotions [23]. In line with this, the inability of a woman with total facial nerve paralysis and normal intelligence to perform a facial expression recognition task was previously reported [24]. Then again, in another study, patients with facial nerve paralysis made at least three more incorrect judgments than the mean of the healthy controls in a facial expression recognition task but had no significant impairment [25]. In contrast, a case study of a woman with total facial paralysis showed no impairment in facial expression experience and recognition [23]. Even with more patients, 18 adults with total facial paralysis, another study [26] reported no widespread deficits in the facial expression recognition task.

Overall, these studies provide a wealth of information on proving or rejecting the *necessity hypothesis*. Nonetheless, none of these studies measured the brain signals of patients with facial nerve paralysis and healthy controls in response to emotional stimuli, and to our knowledge, differences between their brain emotional responses have not yet been reported. It is also unclear whether these patients with facial nerve paralysis have different brain frequencies compared to healthy subjects in response to emotional stimuli. In the present study, we investigate this question by applying a machine learning algorithm that classifies one second of brain activities (measured with MEG) belonging to either facial nerve paralysis patients or healthy controls. The selected machine learning technique in this study is the logistic regression with LASSO regularization, which is highly common in the classification of high-dimensional data and also showed high accuracies in the emotion classification studies (e.g., [27,28,29]). Using this method also showed higher accuracies compared to using the other classification methods in emotion classification studies. For instance, Kim and colleagues [27] reported equivalent or better emotion classification performances using logistic regression than related works using the support vector machine (SVM), and naïve Bayes. Moreover, another EEG study [28] found that logistic regression with LASSO regularization had better performance in emotion classification and less over-fitted results compared to only using logistic regression. They also found that their classification performances using logistic regression with LASSO regularization are higher than those reported by other studies using different classifiers like naïve Bayes, Bayes, and SVM. Caicedo and colleagues [29] also studied the classification of high vs. low valence and high vs. low arousal emotional responses of the brain considering EEG signals. They performed logistic regression with LASSO regularization, SVM, and neural network (NN) as classification methods, and they found that using logistic regression with LASSO regularization makes higher accuracies in both arousal and valence categories compared to SVM and NN. In addition to this strong evidence, using LASSO has the advantage of automatic feature selection by setting the regression coefficients of irrelevant predictors to zero, which is often more accurate and interpretable than achieved estimates produced by univariate or stepwise methods [30,31,32]. Hence, we decided to use the logistic regression with LASSO regularization in our study. The classifications are performed based on considering event-related fields (ERFs) and the power spectrums of five brain frequency bands. In addition, we assess the Self-Assessment Manikin (SAM; [33]) test to determine whether there are differences in the experience of different emotions in these two groups of subjects.

## 2. Materials and Methods

In order to classify the brain’s emotional responses of healthy controls and patients with facial nerve paralysis in three categories (pleasant, neutral, and unpleasant), we proposed a methodology, which is described in the following section.

### 2.1. Subjects

Thirty-three subjects participated in the experiment: 17 healthy (11 females; aged 19–33 years; mean age 26.9 years) and 16 patients with facial nerve paralysis (14 females; age 26–65 years; mean age 45.8 years). Patients were recruited from the Department of Otorhinolaryngology of the Jena University Hospital. All subjects had a normal or corrected-to-normal vision, and healthy subjects had no history of neurological and psychiatric disorders. Beck Depression Inventory (BDI) [34] was measured for patients. The results of this inventory and further information about patients can be found in Table 1. All subjects gave their written informed consent, and the details of the study were approved by the local Ethics Committee of the Jena University Hospital (4415-04/15).

### 2.2. Stimuli and Design

The stimuli consisted of 180 color pictures that were selected from the International Affective Picture System (IAPS; [35]), consisting of three emotional categories (60 pictures each): pleasant, neutral, and unpleasant. The pictures were presented on a white screen in front of the subjects (viewing distance about 80 cm) and were divided into three blocks consisting of 20 pictures of each category in a pseudo-randomized order. Each picture was presented for 6000 ms, followed by varying inter-trial intervals between 2000 to 6000 ms. The three blocks were presented successively, and each block was followed by a short break that allowed the subject to relax. Subjects were asked to avoid eye blinks and eye movements during viewing the pictures and remaining motionless as much as possible. The entire recording process lasted about 45 min and was conducted in a magnetically shielded and sound-sheltered room in the bio-magnetic center of the Jena University Hospital.

After the measurement step, all 180 pictures were presented again in the same order as before, and subjects were requested to rate the level of arousal and valence of each picture. Pictures were rated using the Self-Assessment Manikin (SAM; [33]) with a seven-point scale indicating arousal (1 to 7, relaxed to excited) and valence (1 to 7, pleasant to unpleasant) levels. To find the differences between the ratings between patients and healthy controls, the median ratings of all healthy controls over one picture were compared with the median ratings of 14 patients over the same picture using the Wilcoxon rank-sum test. We did not consider the ratings of two patients because they did not fully participate in this step.

### 2.3. Data Acquisition and Preprocessing

MEG recordings were obtained using a 306-channel helmet-shaped Elekta Neuromag MEG system (Vectorview, Elekta Neuromag Oy, Helsinki, Finland), including 204 gradiometers and 102 magnetometers. In this experiment, only the information of the 102 magnetometers was analyzed. The reason for this is that we had a higher signal-to-noise ratio (SNR) when using magnetometers than when using gradiometers in our study. Moreover, since we used the SSS method, which estimates inside components with the 102 magnetometers and 204 magnetometers, taking magnetometers or gradiometers into account would lead to very similar result measures [36,37,38]. To define the Cartesian head coordinate system, a 3D digitizer (3SPACE FASTRAK, Polhemus Inc., Colchester, VT, USA) was used. MEG was digitized to 24 bit at a sampling rate of 1 kHz. All channels were on-line low-pass filtered at 330 Hz and high-pass filtered at 0.1 Hz. MaxFilter Version 2.0.21 (Elekta Neuromag Oy. Finland) using the signal-space separation (SSS) method [39] was applied on raw data with aligning of sensor-level data across all subjects to one reference subject which helped to achieve the same MEG channel positions for all subjects and quantify the robustness of sensors across all subjects. Then, 1000 ms before the stimulus onset and 1500 ms after the stimulus onset were pre-processed. Baseline correction was applied to the first 1000 ms of the epoch. Data were down-sampled to 250 Hz and band-pass filtered (1–80 Hz). Using the independent component analysis (ICA), eye artifacts (EOG), and artifacts caused by magnetic fields of heartbeat (ECG) were removed. Visual detection was used to identify and remove trials that had excessive movement artifacts. Finally, 45 to 55 trials remained for each stimulus category per subject. The artifact-free data were low-pass filtered at 45 Hz to calculate event-related fields (ERFs). Then the power spectrums of MEG data were calculated in five frequency bands: delta (1–4 Hz), theta (5–8 Hz), alpha (9–14 Hz), beta (15–30 Hz), and gamma (31–45 Hz). The entire analysis was performed using the Fieldtrip toolbox [40] and MATLAB 9.3.0 (Mathworks, Natick, MA, USA).

### 2.4. Feature Extraction

The feature sets used in this study can be categorized into two groups: features based on ERFs, and features based on power spectrums. These features and the classification method used in this experiment are explained in detail in the following section.

#### 2.4.1. Features Based on ERFs

We took the mean values of event-related fields power (i.e., ERFs to the power of two) over one-second post-stimulus and all stimuli of each emotion category as observations for each subject. Thus, each subject provided three vector-valued observations: one for pleasant, one for neutral, and one for unpleasant. Each observation incorporates 102 (magnetometers) elements. Combining observations from all 33 subjects to define the feature matrix in one emotion category (e.g., pleasant), we obtained 33 observations with 102 predictors each. Therefore, based on ERF responses, we compiled three feature sets (according to three emotion categories), and each feature set had a dimensionality of 33 × 102.

#### 2.4.2. Features Based on Power Spectrums

To generate features based on power spectrums, we took the mean values of the power spectrum (from a particular frequency band) over one-second post-stimulus and all stimuli of each emotion category as observations for each subject. Thus, each subject provided three vector-valued observations for each frequency band (5 bands): one for pleasant, one for neutral, and one for unpleasant. Each observation incorporates 102 (magnetometers) elements. Therefore, based on power spectrums, we compiled 15 feature sets (according to three emotion categories and five brain frequency bands) with a dimensionality of 33 × 102 each.

### 2.5. Feature Subset Selection and Classification

After extracting features, we had to select a subset of features that are mostly related to the emotion discrimination of these two groups of subjects and apply classification methods for that subset. The reason for selecting a subset is that from the statistical point of view, irrelevant features may decrease the classification accuracy [41]. However, exploring based on the entire feature set and identifying subsets of discriminative features is a very complex and lengthy process. Thus, using effective feature selection methods helps to avoid the accumulation of features that are not discriminative in the least amount of time possible.

Here, we employed regularized logistic regression with the most popular penalty, the least absolute shrinkage, and selection operator (LASSO; [42]), for feature subset selection and classification. LASSO is highly common in the classification of high dimensional data (a large number of predictors and small sample size) because it selects variables by forcing some regression coefficients to zero and provides high classification accuracies [43].

We defined the response variable of the logistic regression by one for healthy controls and zero for patients with facial nerve paralysis. Let $y_{n}\in \;\left\{0,\;1\right\}$ be a vector with N elements, and let $x_{n}$ be associate vectors with M predictors. The probability of being healthy (class 1) for the $n^{th}$ subject is estimated by Equation (1) [43]:

(1) $$\pi_{n\;}=p\left(y_{n}=1|x_{n}\right)=\;\frac{\exp\;(\beta_{0}+\sum_{m=1}^{M}x_{nm}{}^{T}\beta_{m})}{1\;+\;\exp\;(\beta_{0}+\sum_{m=1}^{M}x_{nm}{}^{T}\beta_{m})}\;\;\;\;\;\;\;\;\;n=1,\;2,\;\ldots ,\;N$$

where $\beta_{m}$ and $\;\beta_{0}$ are the regression coefficients and the intercept, respectively. The goal of LASSO regression is to estimate the $\beta_{m}$ and $\;\beta_{0}\;$ which are obtained by Equation (2) [43]:

(2) $${\hat{\beta }}_{LASSO}=\arg\underset{\beta }{\min}\left[-\sum_{n=1}^{N}\left\{y_{n}\ln\left(\pi_{n}\right)+\left(1-y_{n}\right)\ln\left(1-\pi_{n}\right)\right\}+\;\lambda \;\sum_{m=1}^{M}\left|\beta_{m}\right|\right]$$

**Figure 1:**
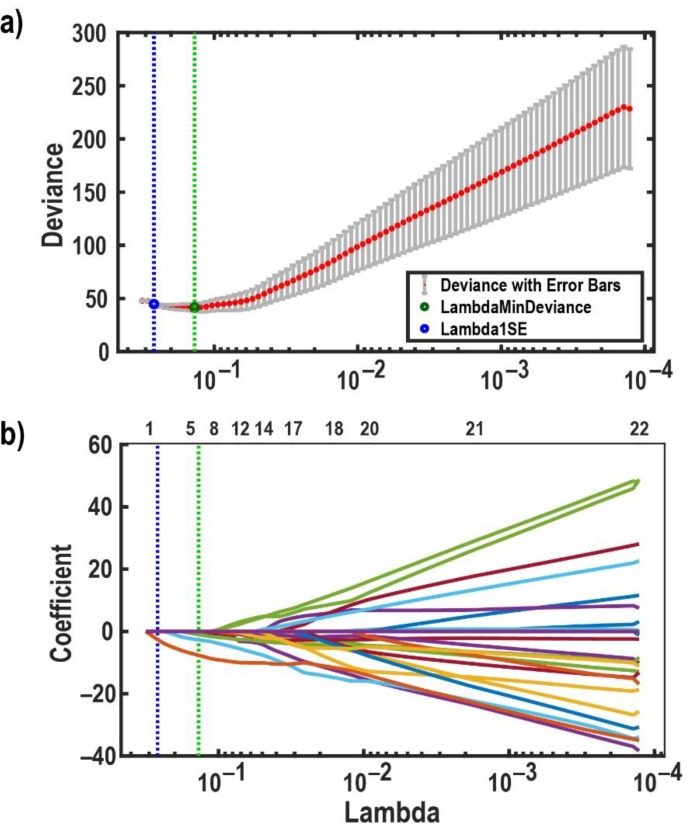
Effects of LASSO regularization tuning parameter λ on regression coefficients, and deviances. (**a**) A plot of the cross-validation deviance of the LASSO fit model against the λ. This figure shows leave-one-subject-out cross-validation results to determine the optimal value of λ. The *Y*-axis indicates the cross-validation deviance corresponds to the values of λ on the *X*-axis. The mean cross-validation deviance is shown by the red points in this figure, and each error bar shows ±1 standard deviation. The blue and green vertical dotted lines (in both figures) indicate the λ, which gives the minimum deviance with no more than one standard deviation (blue circle) and the minimum deviance (green circle), respectively. (**b**) The paths of the LASSO fit model’s coefficients in dependence on λ. This figure shows how λ controls the shrinkage of LASSO coefficients. The numbers above the box show how many non-zero coefficients remain considering the corresponding λ values on the *X*-axis. The *Y*-axis illustrates the coefficients of classifiers. Each path refers to one regression coefficient. It is shown that when λ increases to the left side of the plot, the number of remaining non-zero coefficients gets close to zero.

**Figure 2:**
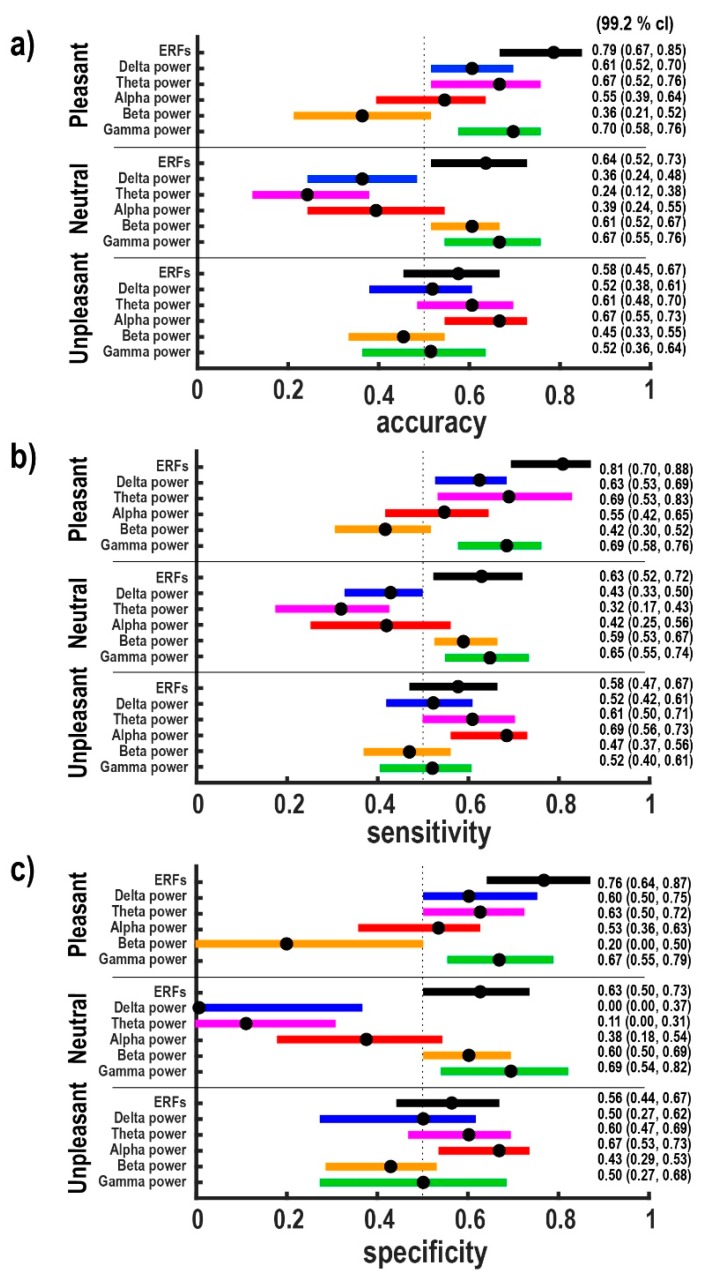
Evaluation of classifier performance for all feature sets based on 1000 16-fold-stratified cross-validations. Numerical values represent medians as well as 95% simultaneous confidence intervals for the metrics (**a**) accuracy, (**b**) sensitivity, and (**c**) specificity. The median values considering 95% CI are represented by circles. The vertical dotted line displays results equal to random results. Considering features based on ERFs in the pleasant category, we achieved the highest classification performances.

The penalty term$,\;\lambda \;\sum_{m=1}^{M}\left|\beta_{m}\right|$, penalizes large regression parameters, where the regularization constant λ, is the positive tuning parameter that controls the balance between the model fit and the effect of the penalty term [44]. When λ=0, maximum likelihood is reached and when  λ tends towards infinity, it increases the impact of the penalty term on the parameter estimates, and the penalty term obliges all regression coefficients to be zero. To determine the optimal value of λ, we used a leave-one-subject-out cross-validation (33-fold). The optimal λ was selected according to the minimum cross-validation error under the constraint that at least two regression coefficients are not equal to zero. This constraint resulted from pilot investigations, which revealed that reliable discrimination of patients and healthy controls was not possible based on univariate features. Since we defined 18 feature sets, we had to determine 18 lambdas; exemplary, we show only one figure related to the lambda determination (see Figure 1). The optimal λ associated with each feature set was used for feature subset selection and classification. The classification performance was evaluated by accuracy, specificity, and sensitivity. The accuracy is the ratio of the correctly classified subjects to their total number. The ratio of correct positives (healthy controls classified as healthy controls) to the total number of healthy controls is called sensitivity or true positive rate. The ratio of correct negatives (patients classified as patients) to the total number of patients is called specificity or true negative rate. To assess our classification results, we performed 1000 16-fold-stratified cross-validations to estimate simultaneous 95%-confidence intervals for accuracy, sensitivity, and specificity (see Figure 2).

## 3. Results

In this section, we assess the feasibility of classifying brain emotional responses of healthy controls and patients with facial nerve paralysis. To this end, we report the classification performances of choosing each feature set. Then we report the results of the comparison between the levels of arousal and valence rated by these two groups of subjects to determine whether there are differences between their experience of emotions or not.

### 3.1. Classification Results

Figure 2a depicts accuracies with 95% simultaneous confidence intervals for all feature sets. Simultaneous confidence intervals were determined at 99.2% of individual confidence levels in order to obtain a 95% simultaneous confidence level using Bonferroni correction for six hypotheses. As can be seen, it is possible to discriminate the brain responses of these two groups of subjects in each category: in the category of pleasant based on ERFs as well as on delta-, theta- and gamma-band power; in the category of neutral based on ERFs as well as on beta- and gamma-band power; in the category of unpleasant based on alpha-band power. The highest accuracy of 0.79 (95% CI (0.70, 0.82), 99.2% CI (0.67, 0.85)) was obtained for pleasant stimuli in combination with a direct exploitation of ERFs. Comparing the three categories, by trend, the groups are best distinguishable for pleasant stimuli.

In order to elaborate that accuracy values are not dominated by one of the subjects’ groups, sensitivities and specificities with 95% simultaneous confidence intervals for all feature sets are presented in Figure 2b,c, respectively. As expected, due to similar group sizes of 16 and 17, statistical significances with respect to sensitivity and specificity resemble the significance pattern of accuracy.

### 3.2. Ratings Results

**Figure 3:**
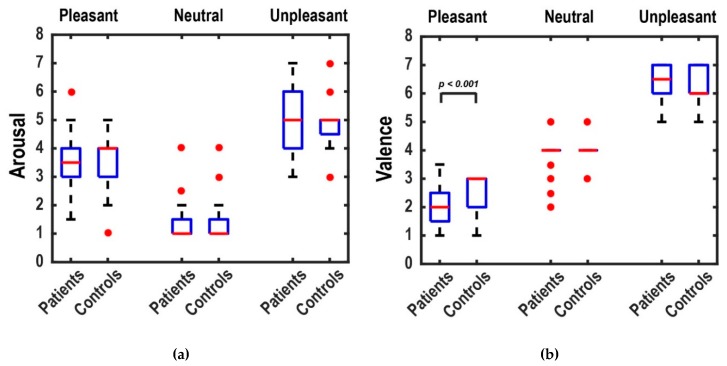
Boxplots of the arousal (**a**) and valence (**b**) ratings of patients and healthy controls for each picture category. Boxplots show the median ratings of subjects for each picture category. The red lines are the medians, and the red circles represent outliers. The valence ratings for pleasant stimuli are significantly higher for healthy controls compared to patients.

The results of the median ratings of patients and healthy controls for arousal and valence levels in three picture categories are indicated in Figure 3. To compare the median ratings between patients and controls, we performed the Wilcoxon rank-sum test, which is based on the null hypothesis of equal medians. We found no significant differences in the comparison of arousal ratings. In the comparison of the valence ratings, we found significant higher valence ratings only for pleasant stimuli in healthy subjects compared to patients ($p=\;1\times 10^{-6}$). This shows that patients rated pleasant images significantly more positively than controls, since, in the 7-point scale of valence, 1 indicates the highest positivity and 7 the highest negativity of an emotion. Except for some outliers, neutral stimuli showed no variation between subjects (median rating = 4).

## 4. Discussion

Facial nerve paralysis is a common disorder of the main motor pathway, which causes an inability to perform facial expressions. In our present study, we investigated the automatic classification of brain responses of patients with facial nerve paralysis and healthy controls using MEG signals in response to three emotional categories of picture stimuli (pleasant, neutral, and unpleasant). We evaluated the feasibility of classifying brain emotional reactions of these two groups of subjects by computing several features based on ERFs and power spectrums in five brain frequency bands. Significant classification performances were obtained for all three emotional categories, and the highest was achieved when considering feature sets taken from ERFs in response to pleasant stimuli with the median of 0.79 (95% CI (0.70, 0.82)). These results demonstrate that patients with facial nerve paralysis might have different emotional brain responses compared to healthy controls. However, comparing the amplitude of brain responses between patients and controls, considering ERFs and power spectra in each frequency band, we found no significant differences. We propose that these differences might relate to the patterns of brain emotional responses. As a physiological explanation, since the loss of movement feedback in facial nerve paralysis influences the cortical motor network [18,19,20,21], which is responsible for the generation of patterned emotion-specific changes in several systems such as the limbic system [45]; it is possible that the produced patterns become different. To the best of our knowledge, there are no studies that report the differences between the brain’s emotional responses of facial nerve paralysis patients and healthy controls. However, our results are consistent with the result of an earlier study that reported blocking facial mimicry of healthy subjects causes different neural activations in the amygdala in response to emotional stimuli [3]. Our results are also compatible with a very recent study that compared patients with facial nerve paralysis and healthy controls in resting state and found that the brain fraction amplitude of low-frequency fluctuation is abnormal in emotion-related regions [46].

Our classification accuracies obtained using logistic regression with LASSO regularization are not compatible with any study because, as mentioned above, to the best of our knowledge, our study is the first to classify the brain’s emotional responses of patients with facial nerve palsy compared to the healthy controls. However, our results can be compatible with the results of emotion classification studies. The accuracies achieved in our study (the highest value: 0.79 (99.2% CI (0.67, 0.85))) are similar or higher than the results achieved with many studies. For instance, an EEG study [47] conducted SVM to classify valence and arousal in human emotions evoked by visual stimuli, and the classification accuracies were between 54.7% and 62.6%. Another EEG study [48] also used SVM to classify four emotion categories (joy, anger, sadness, and pleasure), and the best accuracies were obtained considering joy (86.2%). Using both SVM and the hidden Markov model, one EEG study [49] classified pleasant, unpleasant, and neutral emotion categories, and the highest mean accuracy was 62%. Classification using SVM was also performed in classifying joy, sadness, fear, and relaxed states, which resulted in an average accuracy of 41.7% [50]. Using naïve Bayes and Fisher discriminant analysis (FDA), the average accuracy of 58% was obtained for classifying three arousal categories of picture stimuli by another study [51]. In order to propose Bayesian network-based classifiers in classifying emotions, one EEG study achieved the highest accuracy of 78.17% [52]. To our knowledge, only one study [53] investigated MEG for the classification of human emotions. They performed linear SVM classifiers and achieved the highest classification accuracy of 84%. Our classification accuracies are also similar or higher than the results of other studies using the same classification methods as in our study. For instance, Kim and colleagues [27] reported accuracy of 78.57% performing logistic regression to classify positive versus negative emotions, and they also found that their results were more accurate than results of related works using SVM and naïve Bayes. Another EEG study [28] also reported the maximum accuracy of 78.1% when performing logistic regression with LASSO regularization in the classification of valence and arousal. They also noted that their results achieved by using logistic regression with LASSO regularization were higher than those achieved by logistic regression alone or compared with other studies using classifiers such as naïve Bayes, Bayes, and SVM. Caicedo and colleagues [29] also performed the logistic regression with LASSO regularization to classify arousal and valence. They also used SVM and NN classifiers and achieved an accuracy of 78.2% using logistic regression with LASSO regulation, which was higher than when using SVM and NN.

In our study, the classification accuracies obtained through brain responses to pleasant stimuli were higher than brain responses triggered by other stimuli. This finding indicates that the processing of pleasant emotions in facial nerve paralysis patients are significantly different from those in healthy controls, and this difference is more pronounced than differences between the brain responses evoked by unpleasant or neutral stimuli. However, why might paralysis of facial nerve cause different brain emotional responses to pleasant stimuli, more than to unpleasant stimuli? One possible explanation might be that people control their negative emotions more often than positive emotions [54]. Therefore healthy controls may refrain from performing facial expressions while having unpleasant emotions more than when experiencing pleasant emotions. Thus, there may not be vast differences between the feedback caused by facial expressions during unpleasant stimuli, and consequently, the brain responses triggered by that, in people who can perform (healthy subjects), and people who cannot perform facial expressions (patients with facial nerve paralysis).

In our emotion ratings task, we used the International Affective Picture System (IAPS; [35]), including a wide range of emotional scenes such as nature, war scenes, sports, and family, as opposed to previous studies that contained images of faces [23,24,25,26]. Using the Self-Assessment Manikin (SAM; [33]) test, we demonstrated that patients with facial nerve paralysis significantly rated lower valence levels for pleasant stimuli compared to healthy controls. Since valence is the positivity or the negativity conveyed by an emotion [55], the ratings obtained from subjects in this experiment imply that the effects of pleasant stimuli are more positive for patients than for healthy controls. Thus, our findings demonstrate that facial feedback plays an essential role in the normal experience of pleasant emotional images. This finding is in line with our previous findings regarding the highly significant different brain responses of these two groups of subjects in response to pleasant stimuli, which are reflected in the different experience of pleasant stimuli by these patients. Inconsistent with our results, from the suppression of emotional expressions in healthy controls, Davis and his colleagues [56] demonstrated that inhibiting facial expressions in healthy people makes no difference in positive emotional experiences, but weakens negative emotional experiences. The different emotional experience of patients with facial nerve paralysis and healthy controls has also been reported in earlier studies that evaluated facial expression recognition tasks. Calder and colleagues [25] studied three patients with total facial nerve paralysis and compared their emotion recognition with 40 healthy controls. They reported that patients made at least three times more wrong judgments than the average of the healthy controls, but there was no significant impairment. Giannini and colleagues [24] reported the complete inability of a woman with total facial nerve paralysis in performing a facial expression recognition task. However, some studies found no different emotion recognition by patients with total facial nerve paralysis compared to healthy controls [23,26]. Accordingly, such studies suggest that facial feedback is not necessary to recognize facial expressions, which might oppose the *necessity hypothesis*. Our study does not allow us to support or oppose the previously described *necessity hypothesis* because it requires that we study patients with total facial nerve paralysis. However, our study demonstrates that the paralysis of the facial nerve causes changes in the emotional responses of the brain, especially during pleasant stimuli and these results were also reflected in the ratings of pleasant emotion in these patients. This finding is a strong argument for the importance of the ability to perform facial expressions to have normal brain emotional processing and experience of particular emotions.

Considering different feature sets, we have demonstrated that the brain’s responses to pleasant, unpleasant, and neutral stimuli in patients with facial nerve paralysis are significantly different from those in healthy controls. Moreover, we showed that these different brain responses are associated with the power spectrum of some frequency bands. No study to our knowledge has reported any differences between the frequency-bands of brain emotional responses in the comparison between healthy subjects and patients with facial nerve paralysis. However, we found some biological evidence that may explain some of these results. There is some evidence that gamma-band activity is associated with the activation of the sensorimotor cortex, and gamma event-related synchronization (ERS) occurs in the sensorimotor cortex during unilateral limb movements such as movements of fingers, toes, and tongue [57,58], thus unilateral facial movements might produce gamma activity in the sensorimotor cortex. Since patients with facial nerve paralysis cannot fully perform facial expressions, and because they have impaired connectivity within the sensorimotor cortex [18,19,20,21], their induced gamma activity might be different. Many recent experiments have also focused on the role of theta–gamma oscillations in cognitive neuroscience [59,60,61,62,63]. It is assumed that theta–gamma interactions are associated with cortical sensory processing [64], and a hierarchy of oscillations including delta, theta, and gamma oscillations arranges sensory functions [65]. Thus, impaired connectivity within the sensorimotor cortex caused by facial nerve paralysis can lead to a disruption of the delta–theta–gamma oscillations. Moreover, it has been demonstrated that when sensory and motor regions become engaged, the suppression of alpha power has been observed [63]. Given that the facial nerve is mainly associated with the primary sensorimotor area [16,17] and the paralysis of that results in reduced connectivity within the cortical facial motor network [18,19,20,21], it might be the reason for different alpha power in patients with paralysis of facial nerve compared to healthy controls.

Finding significant classification accuracies in the classification of brain neutral responses of these two groups of subjects were interesting, unexpected findings. Since it is assumed that neutral stimuli have non-emotional content, we did not expect to find different brain responses of these two groups of subjects during viewing neutral stimuli. However, we have shown that the brain ERFs of patients with facial nerve paralysis differ significantly from those of healthy controls and are associated with beta and gamma bands. Nonetheless, one possibility might be that mood changes when viewing affective pictures may influence the processing of neutral pictures, and the presentation of neutral images alone may lead to more accurate results [66].

## 5. Limitations in the Study

There are some limitations in this study that should be considered in further research. First, the mean age of patients in this study was greater than in healthy subjects. This was because most facial nerve paralysis patients were not interested in participating in the experiment. Therefore, we did not have many possibilities to consider the same mean age for both groups of participants. The reason for the unwillingness of these patients to participate in such studies might be that they feel reluctant to communicate with other people or to participate in public [67,68]. However, it might be beneficial to consider the same mean age in both groups of subjects in further research. The second issue that could be considered in future studies is to include other emotional categories such as anger, anxiety, or surprise to better classify all emotional states of these two groups of people. Third, in this study, we did not measure the brain activations of these two groups of subjects during the resting state. We would suggest that considering the data of the resting state in further studies would help to find the answer to these very important questions, namely whether these two groups of subjects have different brain activities even at baseline or whether some of the brain rhythms reflect the differences between these two groups of test subjects at baseline.

## 6. Conclusions

This study shows that the emotional experiences and the brain’s emotional responses of patients with facial nerve paralysis are accurately separated from those in healthy controls in specific emotions. Our results suggest that the ability to perform facial expressions is necessary to have normal emotional processing and experience of emotions.

## Figures and Tables

**Table 1:** Characteristics of the facial nerve paralysis patients in this study.

Patient Number.	Gender ^1^	Side	Duration of Having Facial Paralysis in Month	Degree of Paralysis	Type of Paralysis	Reason for Facial Paralysis ^2^	Becks Depression Inventory (BDI)	Depression’s Severity According to BDI
1	W	Left	72	Complete	Chronic	3	10	Mild
2	W	Right	58	Complete	Chronic	1	12	Mild
3	W	Right	101	Complete	Chronic	1	1	Minimal
4	W	Left	40	Complete	Chronic	1	44	Severe
5	W	Left	29	Complete	Chronic	1	25	Moderate
6	W	Right	79	Complete	Chronic	1	3	Minimal
7	M	Left	35	Complete	Acute	2	6	Minimal
8	W	Right	23	Complete	Acute	1	7	Minimal
9	W	Right	71	Complete	Chronic	1	8	Minimal
10	W	Left	25	Complete	Chronic	1	13	Mild
11	W	Right	22	Complete	Chronic	2	3	Minimal
12	W	Right	21	Complete	Acute	1	17	Mild
13	M	Right	17	Complete	Chronic	1	2	Minimal
14	W	Left	99	Complete	Chronic	3	6	Minimal
15	W	Left	19	Complete	Acute	2	4	Minimal
16	W	Left	16	Complete	Chronic	3	15	Mild

^1^ W: woman, M: man, ^2^ 1 = idiopathic, ^2^ = inflammation, ^3^ = post-surgical.
